# Additions to Pleosporalean Taxa Associated with *Xanthoceras sorbifolium* from Jilin and Hebei, China

**DOI:** 10.3390/microorganisms13061296

**Published:** 2025-05-31

**Authors:** Rong Xu, Yu Li

**Affiliations:** 1 Engineering Research Center Edible and Medicinal Fungi, Ministry of Education, Jilin Agricultural University, Changchun 130118, China; zyxurong66@126.com; 2 School of Food Science and Engineering, Yangzhou University, Yangzhou 225127, China

**Keywords:** *Pleosporales*, taxonomy, saprobic fungi, *Xanthoceras sorbifolium*, phylogeny

## Abstract

Pleosporalean fungi play significant roles as plant pathogens, saprobes, and endophytes in a wide variety of economically important plant hosts. During an investigation of saprobic fungi from Jilin and Hebei, China, five pleosporalean isolates were obtained from the dead stems of *Xanthoceras sorbifolium*. Morphological evidence and multi-locus sequence analyses using a combined dataset of ITS, LSU, SSU, *rpb*2, *tef*1-α, and *tub*2 indicate that these isolates represent two new species (*Alloleptosphaeria xanthoceratis* and *Lophiostoma multiforme*) and a new record of *Lophiostoma montanae*. Full morphological descriptions and illustrations are provided herein, and phylogenetic relationships of three pleosporalean taxa are also discussed. Detailed morphological descriptions and illustrations are presented, along with phylogenetic affiliations of three pleosporalean taxa.

## 1. Introduction

*Ascomycota*, commonly known as sac fungi, constitutes the largest and most ecologically diverse phylum within the fungal kingdom [1]. The phylum exhibits remarkable morphological variability, ranging from unicellular yeasts to multicellular structures producing elaborate fruiting bodies [2,3]. They occupy virtually every terrestrial and aquatic habitat, functioning as decomposers, pathogens, endophytes, and mutualistic symbionts (e.g., lichens and mycorrhizae) [4]. These fungi are economically important as antibiotic producers, industrial agents, model organisms, and sources of bioactive metabolites [5]. Given their ecological dominance and biotechnological relevance, *Ascomycota* remain a critical focus of mycological research.

*Pleosporales*, a diverse order in the class *Dothideomycetes* (*Ascomycota*), comprises over 10,000 species across more than 90 families [6]. Members of this order are characterized by pseudothecial ascomata, bitunicate and fissitunicate asci, and aseptate or septate ascospores that vary in pigmentation, shape, and gelatinous sheath [7]. Asexual morphs are predominantly coelomycetous or can sometimes be hyphomycetous [8,9,10,11]. Pleosporalean fungi contain saprobic, pathogenic, endophytic, parasitic, hyperparasitic, and lichenized species that have successfully colonized virtually all global habitats, from terrestrial to aquatic environments [7,8,9]. Over the past decade, molecular phylogenetic studies coupled with morphological evidence have significantly refined the taxonomy of *Pleosporales*, revealing abundant diversity and leading to the establishment of new families and genera [6,12]. Recent investigations in China have revealed numerous novel species associated with woody oil plants [13,14,15].

*Xanthoceras sorbifolium* (*Sapindaceae*) is an important tree native to northern China with resistance to drought and could be used for windbreak, sand fixation, and desertification control [16,17]. It also has antitumor and anti-inflammatory activities [18]. Notably, its oil-rich seeds (containing >50% unsaturated fatty acids) have garnered attention as a promising biodiesel feedstock and edible oil source with potential cardiovascular benefits [18]. While extensively studied for its medicinal properties, oil-rich seeds, and stress tolerance, the microfungi associated with this plant remain underexplored despite their potential agricultural and ecological implications [18]. Preliminary investigations have identified several saprobic and pathogenic fungi, including *Angularia xanthoceratis* [19], *Comoclathris xanthoceratis* [20], *Neocucurbitaria subribicola* [21], *Fusarium* sp., and *Verticillium* sp. [22], which reflect the abundance of potential microfungal resources in *X. sorbifolium*. 

During a survey of microfungi allied with *X. sorbifolium* in China, a series of interesting pleosporalean fungi was collected. This study aimed to enrich knowledge of the pleosporalean taxa in *X. sorbifolium*. Morphological comparisons integrated with multilocus phylogenetic analyses were conducted to determine the classification of these new collections. Two novel species and one newly recorded species are described from Jilin and Hebei Provinces, China.

## 2. Materials and Methods

### 2.1. Sample Collection, Isolation, and Morphological Observation

During a series of fieldwork from 2021 to 2022 in Jilin and Hebei Provinces, China, dried woody litter samples were collected. The collected samples, labeled with location, date, host, and collection data in plastic bags, were transported to the lab for morphological study. Pure colonies were obtained through single spore isolation [23] and incubated on potato dextrose agar (PDA) at 25 °C for 2 to 6 weeks. The specimens were preserved at the Herbarium of Mycology, Jilin Agricultural University (HMJAU), Changchun, China, while the pure cultures were stored in the Culture Collection of the International Cooperation Research Center of China for New Germplasm Breeding of Edible Mushrooms (CCMJ). The novel species were documented and assigned accession numbers in MycoBank [24].

Fungal morphological structures were examined and photographed using a Zeiss Stemi 2000C stereo microscope (paired with a Leica DFC450C camera) and a Zeiss AX10 light microscope (fitted with an Axiocam 506 camera) (ZEISS/Leica, Jena/Wetzlar, Germany). Microscopic elements were measured using the ZEN 3.4 (blue edition) program (ZEISS, Jena, Germany), and all images were processed with Adobe Photoshop 2020 (Adobe Systems, San Jose, CA, USA).

### 2.2. DNA Extraction, PCR Amplification, and Sequencing

Fungal genomic DNA was extracted from mycelium using the NuClean PlantGen DNA Kit (CWBIO, Taizhou, China) following the manufacturer’s protocol. The DNA amplification was conducted by polymerase chain reaction (PCR) using internal transcribed spacers (ITS), large subunit (LSU) rDNA, small subunit (SSU) rDNA, translation elongation factor 1-α (*tef*1-α), RNA polymerase II second largest subunit (*rpb*2), and beta-tubulin (*tub*2). The specific primer pairs for six molecular markers are described in Table 1. Amplification was conducted following Xu et al. [19,21], with PCR products confirmed via 1% agarose gel electrophoresis (stained with 0.5 mL of 10,000× DNA dye; Biotium, Fremont, CA, USA). Successful amplifications were sequenced by Sangon Biotech (Shanghai, China). The newly obtained sequences have been submitted to GenBank [25], with accession numbers provided in Table 2 and Table 3.

### 2.3. Molecular Phylogeny

The new strains were initially identified by molecular techniques through comparison of individual gene sequences using BLASTn [31], with relevant sequence data acquired from GenBank (Table 2 and Table 3). Alignments were generated using MAFFT v.7 [32] with the L-INS-i algorithm (1PAM/k = 2 scoring matrix, 1.50 gap opening penalty for nucleotide sequences), followed by manual adjustment in BioEdit v7.2.5 [33]. The AliView program was used to convert the alignment data files to PHYLIP and NEXUS formats [34]. 

**Figure 1:**
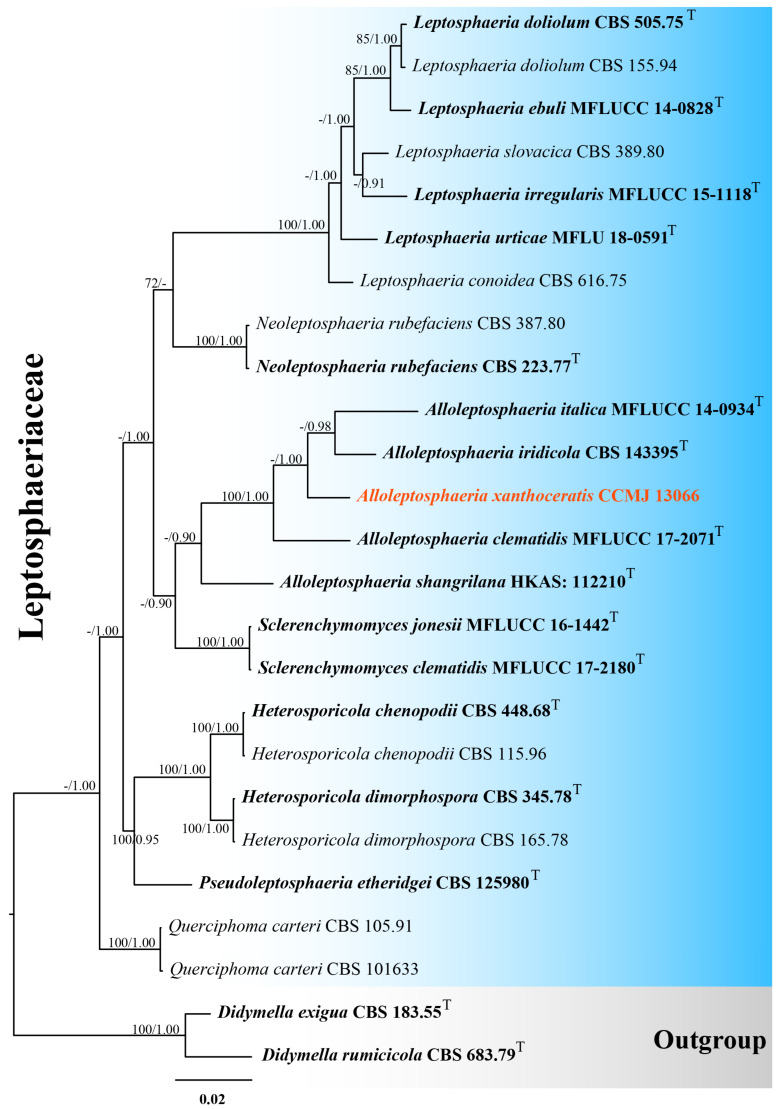
The Bayesian 50% majority-rule consensus tree based on concatenated ITS, LSU, SSU, and *tub*2 sequences in *Leptosphaeriaceae*. The tree is rooted with *Didymella exigua* (CBS 183.55) and *D. rumicicola* (CBS 683.79). Maximum likelihood bootstrap support values ≥ 70% (ML) and Bayesian posterior probabilities ≥ 0.9 (BPP) are given at the nodes as ML/BPP. The type strains are in bold and black. The newly generated isolates are shown in bold red and marked with “T”.

**Figure 2:**
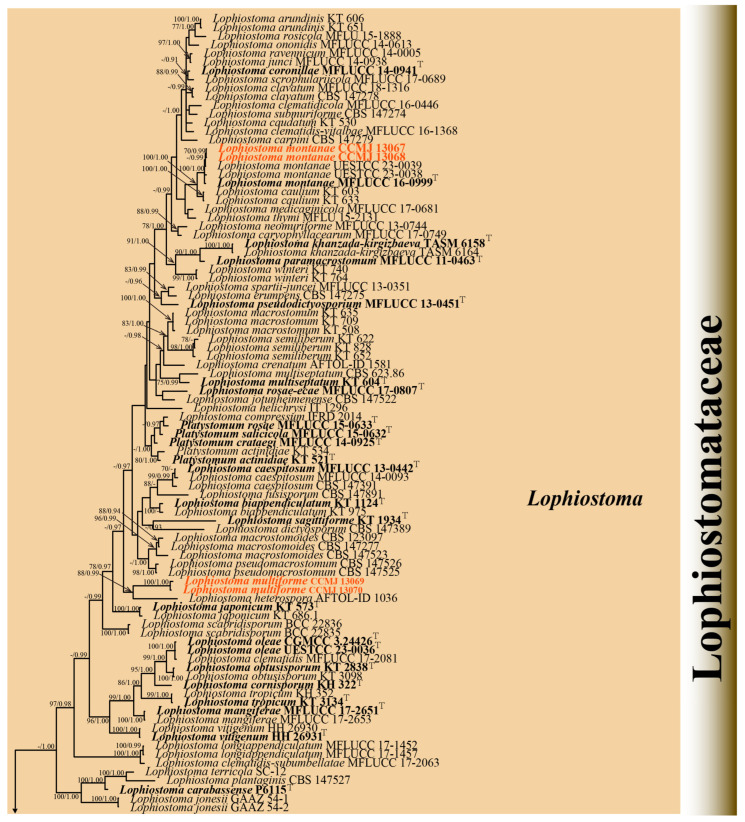

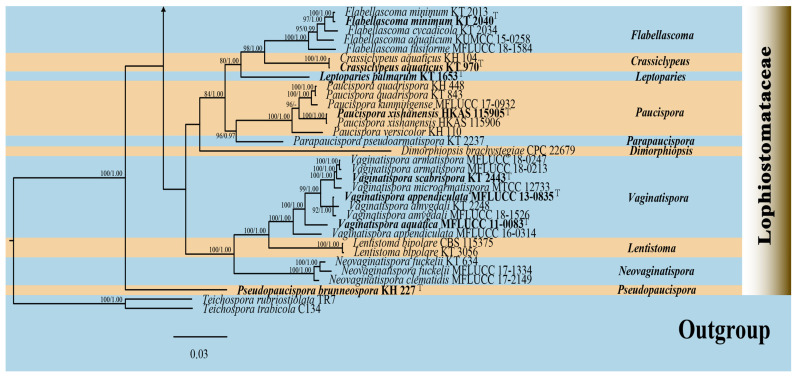
The Bayesian 50% majority-rule consensus tree based on concatenated ITS, LSU, SSU, *tef*1-α, and *rpb*2 sequences in *Lophiostomataceae*. The tree is rooted with *Teichospora rubriostiolata* (TR7) and *T. trabicola* (C134). Maximum likelihood bootstrap support values ≥ 70% (ML) and Bayesian posterior probabilities ≥ 0.9 (BPP) are at the nodes as ML/BPP. The type strains are in bold and black. The newly generated isolates are indicated in bold red and marked with “T”.

Phylogenetic trees were constructed using individual genetic markers and subsequently analyzed in combination, along with a concatenated multi-gene dataset. Maximum likelihood analysis was conducted with RAxML-HPC2 on XSEDE through the CIPRES web portal (http://www.phylo.org/portal2/; accessed 28 March 2025) [35]. The optimal evolutionary models for both individual and concatenated datasets were determined using jModeltest 2.1.10, with model selection based on the Akaike Information Criterion (AIC) for posterior probability analysis [36]. The GTR+GAMMA model of nucleotide evolution was applied to all datasets with 1000 bootstrap repeats. BI analysis was performed using MrBayes v.3.2.6 implemented through the CIPRES Science Gateway portal (https://www.phylo.org; accessed on 29 March 2025) [37]. Simultaneous Markov chains were run for 800 million generations. Trees were sampled every 100 generations, with the first 20% discarded as burn-in. Outgroup taxa selection comprised *Didymella exigua* (CBS 183.55), *D. rumicicola* (CBS 683.79), *Teichospora rubriostiolata* (TR7), and *T. trabicola* (C134) (Figure 1 and Figure 2). The phylogenetic tree file was downloaded from CIPRES and visualized in FigTree v.1.4.4 [38]. The tree designs were crafted in Adobe Illustrator CS6.

**Table 2:** Names and corresponding GenBank accession numbers of *Leptosphaeriaceae* taxa used in phylogenetic analyses, with new sequences in bold blue and type strains in bold.

Species	Strain/Isolate	GenBank Accession Numbers			
		ITS	LSU	SSU	*tub*2
***Alloleptosphaeria italica***	**MFLUCC 14-0934**	**KT454722**	**KT454714**	_	_
***A. clematidis***	MFLUCC 17-2071	MT310604	MT214557	MT226674	_
***A. iridicola***	CBS 143395	MH107919	MH107965	_	NA
***A. shangrilana***	HKAS:112210	MW431059	MW431315	MW431058	NA
***A. xanthoceratis***	**CCMJ 13066**	**PP151694**	**PP153449**	**PV569760**	**PV670045**
***Didymella exigua***	**CBS 183.55**	**GU237794**	**EU754155**	**EU754056**	**GU237525**
***D. rumicicola***	**CBS 683.79**	**KT389503**	**KT389721**	_	**KT389800**
***Heterospora chenopodii***	**CBS 448.68**	**FJ427023**	**EU754187**	**EU754088**	_
*H. chenopodii*	CBS 115.96	JF740227	EU754188	EU754089	_
*H. dimorphospora*	CBS 165.78	JF740204	JF740281	JF740098	_
***H. dimorphospora***	**CBS 345.78**	**JF740203**	**GU238069**	**GU238213**	_
*Lep. conoidea*	CBS 616.75	JF740201	JF740279	_	KT389804
*Lep. doliolum*	CBS 155.94	JF740207	JF740282	_	JF740146
***Lep. doliolum***	**CBS 505.75**	**JF740205**	**GQ387576**	**GQ387515**	JF740144
***Lep. ebuli***	**MFLUCC 14-0828**	**KP744446**	**KP744488**	**KP753954**	_
***Lep. irregularis***	MFLUCC 15-1118	KX856056	KX856055	_	_
*Lep. slovacica*	CBS 389.80	JF740247	JF740315	JF740101	_
***Lep. urticae***	MFLU 18-0591	MK123333	MK123332	MK123329	_
***Neoleptosphaeria jonesii***	MFLUCC 16-1442	KY211869	KY211870	KY211871	_
***Neol. rubefaciens***	**CBS 223.77**	**JF740243**	**JF740312**	_	_
*Neol. rubefaciens*	CBS 387.80	JF740242	JF740311	_	_
***Pseudoleptosphaeria etheridgei***	**CBS 125980**	**JF740221**	**JF740291**	_	_
*Querciphoma carteri*	CBS 105.91	KF251209	GQ387594	GQ387533	KF252700
*Q. carteri*	CBS 101633	KF251210	GQ387593	GQ387532	KF252701
*Sclerenchymomyces clematidis*	MFLUCC 17-2180	MT310605	MT214558	MT226675	_

**Table 3:** Names and corresponding GenBank accession numbers of *Lophiostomataceae* taxa used in phylogenetic analyses, with new sequences in bold blue and type strains in bold.

Species	Strain/Isolate	GenBank Accession Numbers				
		ITS	LSU	SSU	*tef*1-α	*rpb*2
*Crassiclypeus aquaticus*	KH 104	LC312499	LC312528	LC312470	LC312557	LC312586
***C. aquaticus***	**KT 970**	**LC312501**	**LC312530**	**LC312472**	**LC312559**	**LC312588**
*Dimorphiopsis brachystegiae*	CPC 22679	KF777160	KF777213	_	_	_
*Flabellascoma aquaticum*	KUMCC 15-0258	MN304827	MN274564	MN304832	MN328898	MN328895
*F. cycadicola*	KT 2034	LC312502	LC312531	LC312473	LC312560	LC312589
*F. fusiforme*	MFLUCC 18-1584	MN304830	MN274567	_	MN328902	_
*F. minimum*	KT 2013	LC312503	LC312532	LC312474	LC312561	LC312590
***F. minimum***	**KT 2040**	**LC312504**	**LC312533**	**LC312475**	**LC312562**	**LC312591**
*Lentistoma bipolare*	KT 3056	LC312513	LC312542	LC312484	LC312571	LC312600
*Len. bipolare*	CBS 115375	LC312506	LC312535	LC312477	LC312564	LC312593
***Leptoparies palmarum***	**KT 1653**	**LC312514**	**LC312543**	**LC312485**	**LC312572**	**LC312601**
*Lophiostoma arundinis*	KT 606	JN942964	AB618998	AB618679	LC001737	JN993482
*Lop. arundinis*	KT 651	JN942965	AB618999	AB618680	LC001738	JN993486
*Lop. biappendiculatum*	KT 975	_	GU205228	GU205254	_	_
***Lop. biappendiculatum***	**KT 1124**	_	**GU205227**	**GU205256**	_	_
*Lop. caespitosum*	CBS 147391	MW759252	MW750387	_	MW752404	MW752383
***Lop. caespitosum***	**MFLUCC 13-0442**	**KP899134**	**KP888639**	**KP899125**	**KR075161**	_
*Lop. caespitosum*	MFLUCC 14-0993	KP899135	KP888640	KP899126	KR075162	_
***Lop. carabassense***	**CBS 149324**	**MT679671**	**OL544969**	**OL544968**	**OL554876**	_
*Lop. carpini*	CBS 147279	MW759258	MW750386	_	MW752405	MW752384
*Lop. caryophyllacearum*	MFLUCC 17-0749	MG828964	MG829076	MG829176	MG829238	_
*Lop. caudatum*	KT 530	LC001723	AB619000	AB618681	LC001739	_
*Lop. caulium*	KT 603	LC001724	AB619001	AB618682	LC001740	_
*Lop. caulium*	KT 633	LC001725	AB619002	AB618683	LC001741	_
*Lop. clavatum*	CBS 147278	MW759259	MW750385	_	MW752406	MW752385
*Lop. clavatum*	MFLUCC 18-1316	_	MN274566	MN304835	MN328901	_
*Lop. clematidicola*	MFLUCC 16-0446	MT310609	MT214563	MT226680	MT394742	_
*Lop. clematidis*	MFLUCC 17-2081	MN393004	MT214562	MT226679	MT394741	MT394689
*Lop. clematidis-subumbellatae*	MFLUCC 17-2063	MT310607	MT214560	MT226677	MT394739	MT394687
*Lop. clematidis-vitalbae*	MFLUCC 16-1368	MT310610	MT214564	MT226681	MT394743	_
*Lop. compressum*	CBS 147276	MW759272	MW750382	_	MW752408	MW752381
*Lop. compressum*	IFRD 2014	_	FJ795437	FJ795480	_	FJ795457
***Lop. cornisporum***	**KH 322**	**LC312515**	**LC312544**	**LC312486**	**LC312573**	**LC312602**
***Lop. coronillae***	**MFLUCC 14-0941**	**KT026120**	**KT026112**	**KT026116**	_	_
*Lop. crenatum*	AFTOL-ID 1581	_	DQ678069	DQ678017	DQ677912	DQ677965
*Lop. dictyosporum*	CBS 147389	MW759251	MW750379	_	MW752411	MW752388
*Lop. erumpens*	CBS 147275	MW759262	MW750381	_	MW752409	MW752386
*Lop. fusisporum*	CBS 147891	MW759253	_	_	MW752401	MW752382
*Lop. helichrysi*	IT-1296	KT333435	KT333436	KT333437	KT427535	_
*Lop. heterosporum*	AFTOL-ID 1036	GQ203795	AY016369	_	DQ497609	DQ497615
*Lop. japonicum*	KT 686-1	LC001729	AB619006	AB618687	LC001745	_
***Lop. japonicum***	**KT 573**	**LC001728**	**AB619005**	**AB618686**	**LC001744**	_
*Lop. jonesii*	GAAZ 54-1	KX687757	KX687753	KX687755	KX687759	_
*Lop. jonesii*	GAAZ 54-2	KX687758	KX687754	KX687756	KX687760	_
*Lop. jotunheimenense*	CBS 147522	MW759261	MW750394	_	MW752392	_
*Lop. junci*	MFLUCC 14-0938	MG828966	MG829078	MG829178	NA	_
***Lop. khanzada-kirgizbaeva***	**TASM 6158**	**MZ966265**	**OK017520**	**OK017525**	**MZ997338**	_
*Lop. khanzada-kirgizbaeva*	TASM 6164	MZ966266	OK017521	OK017526	MZ997339	_
*Lop. longiappendiculatum*	MFLUCC 17-1452	MT214368	MT214462	MT214415	MT235783	_
*Lop. longiappendiculatum*	MFLUCC 17-1457	MT214369	MT214463	MT214416	MT235784	MT235821
*Lop. macrostomoides*	CBS 147523	MW759256	MW750389	_	_	_
*Lop. macrostomoides*	CBS 147277	MW759257	MW750384	_	MW752407	MW752380
*Lop. macrostomoides*	CBS 123097	_	FJ795439	FJ795482	GU456277	FJ795458
*Lop. macrostomum*	KT 508	JN942961	AB619010	_	LC001751	JN993491
*Lop. macrostomum*	KT 709/HHUF 27293	AB433276	AB433274	AB521732	LC001753	JN993493
*Lop. macrostomum*	KT 635/HHUF 27290	AB433275	AB433273	AB521731	LC001752	JN993484
***Lop. mangiferae***	**MFLUCC 17-2651**	**MG931031**	**MG931025**	**MG931028**	_	_
*Lop. mangiferae*	MFLUCC 17-2653	MG931032	MG931026	MG931029	_	_
*Lop. medicaginicola*	MFLUCC 17-0681	MG828967	MG829079	MG829179	_	_
***Lop. montanae***	**MFLUCC 16-0999**	**MT310611**	**MT214565**	**MT226682**	**MT394744**	_
*Lop. montanae*	UESTCC 23.0038	OR253137	OR253296	OR253209	OR263570	OR253750
*Lop. montanae*	UESTCC 23.0039	OR253138	OR253297	OR253210	OR251148	OR253751
***Lop. montanae***	**CCMJ 13067**	**PV569791**	**PV569911**	**PV569764**	**PV670048**	**PV670046**
***Lop. montanae***	**CCMJ 13068**	**PV569792**	**PV569912**	**PV569765**	**PV670049**	**PV670047**
***Lop. multiforme***	**CCMJ 13069**	**PP151705**	**PP153460**	**PV569761**	**PV670043**	**PV670041**
***Lop. multiforme***	**CCMJ 13070**	**PP151706**	**PP153461**	**PV569762**	**PV670044**	**PV670042**
*Lop. multiseptatum*	CBS 623.86	_	GU301833	GU296163	_	GU371791
***Lop. multiseptatum***	**KT 604**	**LC001726**	**AB619003**	**AB618684**	**LC001742**	_
*Lop. neomuriforme*	MFLUCC 13-0744	KY496740	KY496719	KY501110	_	_
*Lop. obtusisporum*	KT 3098	LC312519	LC312548	LC312490	LC312577	LC312606
***Lop. obtusisporum***	**KT 2838**	**LC312518**	**LC312547**	**LC312489**	**LC312576**	**LC312605**
***Lop. oleae***	**CGMCC 3.24426**	**OR253081**	**OR253233**	**OR253172**	**OR262141**	**OR262130**
***Lop. oleae***	**UESTCC 23.0036**	**OR253079**	**OR253231**	**OR253171**	**OR262139**	**OR262129**
*Lop. ononidis*	MFLUCC 14-0613	KU243128	KU243125	KU243126	KU243127	_
***Lop. paramacrostomum***	**MFLUCC 11-0463**	_	**KP888636**	**KP899122**	_	_
*Lop. plantaginis*	CBS 147527	MW759250	MW750378	_	_	MW752375
***Lop. pseudodictyosporium***	**MFLUCC 13-0451**	**KR025858**	**KR025862**	_	_	_
*Lop. pseudomacrostomum*	CBS 147525	MW759255	MW750391	_	MW752395	_
*Lop. pseudomacrostomum*	CBS 147526	MW759254	MW750392	_	MW752394	_
*Lop. ravennicum*	MFLUCC 14-0005	KP698413	KP698414	KP698415	_	_
***Lop. rosae-ecae***	**MFLUCC 17-0807**	**MG828924**	**MG829033**	**MG829139**	**MG829217**	_
*Lop. rosicola*	MFLU 15-1888	MG828968	MG829080	MG829180	MG829240	_
***Lop. sagittiforme***	**KT 1934**	**AB369268**	**AB369267**	**AB618693**	**LC001756**	_
*Lop. scabridisporum*	BCC 22835	_	GQ925844	GQ925831	GU479857	GU479830
*Lop. scabridisporum*	BCC 22836	_	GQ925845	GQ925832	GU479856	GU479829
*Lop. scrophulariicola*	MFLUCC 17-0689	MG828969	MG829081	_	_	_
*Lop. semiliberum*	KT 622	JN942966	AB619012	AB618694	LC001757	JN993483
*Lop. semiliberum*	KT 652	JN942967	AB619013	AB618695	LC001758	JN993485
*Lop. semiliberum*	KT 828	JN942970	AB619014	AB618696	LC001759	JN993489
*Lop. spartii-juncei*	MFLUCC 13-0351	KP899136	KP888641	KP899127	KR075163	_
*Lop. submuriforme*	CBS 147274	MW759260	MW750380	_	MW752410	MW752387
*Lop. terricola*	SC-12	JN662930	JX985750	JX985749	_	_
*Lop. thymi*	MFLU 15-2131	MG828970	MG829082	MG829182	MG829241	_
*Lop. tropicum*	KH 352	LC312521	LC312550	LC312492	LC312579	LC312608
***Lop. tropicum***	**KT 3134**	**LC312522**	**LC312551**	**LC312493**	**LC312580**	**LC312609**
*Lop. vitigenum*	HH 26930	LC001735	AB619015	AB618697	LC001761	_
***Lop. vitigenum***	**HH 26931**	**LC001736**	**AB619016**	**AB618698**	**LC001762**	_
*Lop. winteri*	KT 740	JN942969	AB619017	AB618699	LC001763	JN993487
*Lop. winteri*	KT 764	JN942968	AB619018	AB618700	LC001764	JN993488
*Neovaginatispora clematidis*	MFLUCC 17-2149	MT310606	MT214559	MT226676	MT394738	_
*Neov. fuckelii*	MFLUCC 17-1334	MN304828	MN274565	MN304833	MN328899	MN328896
*Neov. fuckelii*	KT 634	LC001732	AB619009	AB618690	LC001750	_
*Parapaucispora pseudoarmatispora*	KT 2237	LC100021	LC100026	LC100018	LC100030	_
*Paucispora kunmingense*	MFLUCC 17-0932	MF173432	MF173428	MF173430	MF173434	MF173436
*Pa. quadrispora*	KH 448	LC001733	LC001722	LC001720	LC001754	_
*Pa. quadrispora*	KT 843	LC001734	AB619011	AB618692	LC001755	_
*Pa. versicolor*	KH 110	AB918731	AB918732	LC001721	LC001760	_
***Pa. xishanensis***	**HKAS 115905**	**MZ966267**	**OK017522**	**OK017527**	**MZ997340**	_
*Pa. xishanensis*	HKAS 115906	MZ966268	OK017523	OK017528	MZ997341	_
***Platystomum actinidiae***	**KT 521**	**JN942963**	**JN941380**	**JN941375**	**LC001747**	**JN993490**
*Pl. actinidiae*	KT 534	JN942962	JN941379	JN941376	LC001748	JN993492
***Pl. crataegi***	**MFLUCC 14-0925**	**KT026117**	**KT026109**	**KT026113**	**KT026121**	_
***Pl. rosae***	**MFLUCC 15-0633**	**KT026119**	**KT026111**	**KT026115**	_	_
***Pl. salicicola***	**MFLUCC 15-0632**	**KT026118**	**KT026110**	**KT026114**	_	_
***Pseudopaucispora brunneospora***	**KH 227**	**LC312523**	**LC312552**	**LC312494**	**LC312581**	**LC312610**
*Vaginatispora amygdali*	KT 2248	LC312524	LC312553	LC312495	LC312582	LC312611
*V. amygdali*	MFLUCC 18-1526	MK085055	MK085059	MK085057	MK087657	_
*V. appendiculata*	MFLUCC 16-0314	KU743217	KU743218	KU743219	KU743220	_
***V. appendiculata***	**MFLUCC 13-0835**	_	**KY264745**	**KY264749**	_	_
***V. aquatica***	**MFLUCC 11-0083**	**KJ591577**	**KJ591576**	**KJ591575**	_	_
*V. armatispora*	MFLUCC 18-0247	MK085056	MK085060	MK085058	MK087658	MK087669
*V. armatispora*	MFLUCC 18-0213	MN304826	MN274563	MN304831	MN328897	MN328894
*V. microarmatispora*	MTCC 12733	MF142592	MF142593	MF142594	MF142595	MF142596
***V. scabrispora***	**KT 2443**	**LC312525**	**LC312554**	**LC312496**	**LC312583**	**LC312612**
*Teichospora rubriostiolata*	TR7	KU601590	_	_	KU601609	KU601599
*T. trabicola*	C134	KU601591	_	_	KU601601	KU601600

## 3. Results

### 3.1. Phylogenetic Analyses

#### 3.1.1. Phylogenetic Analyses of *Alloleptosphaeria*

One strain was successfully isolated in the laboratory. Phylogenetic analyses incorporated 25 strains using a 2801-character concatenated alignment (ITS: 556 bp; LSU: 881 bp; SSU: 1023 bp; *tub*2: 341 bp; gaps included). The optimal RAxML tree (likelihood score: −8462.532314) was derived from an alignment of 504 distinct patterns, including 25.52% undetermined characters/gaps. Estimated base frequencies were as follows: A = 0.244324, C = 0.224019, G = 0.271360, and T = 0.260297; substitution rates, AC = 1.370239, AG = 2.327667, AT = 1.974968, CG = 0.418253, CT = 5.227042, and GT = 1.000000; evolutionary parameters included gamma shape (α = 0.583180) and invariable sites (I = 0.753617). For the Bayesian analysis, a total of 1598 trees were retained after the 20% burn-in with a stop value of 0.008440. Both ML and BI methods yielded consistent topologies (Figure 1 and Figure S1). In our phylogenetic tree, *Alloleptosphaeria xanthoceratis* (CCMJ 13066) formed a distinct lineage with 42% ML and 1.00 BPP support (Figure 1).

#### 3.1.2. Phylogenetic Analyses of *Lophiostoma*

Four strains were successfully isolated in the laboratory. Phylogenetic analyses were performed using a concatenated alignment of ITS (1–603 bp), LSU (604–1521 bp), SSU (1522–2503 bp), *tef*1-α (2504–3468 bp), and *rpb*2 (3469–4494 bp) sequences from 125 strains, totaling 4494 characters (including gaps). The RAxML analysis yielded a best-scoring tree (Figure 2) with a final ML optimization likelihood value of −34137.002757. The matrix had 1885 distinct alignment patterns, with 26.95% of undetermined characters or gaps. Estimated base frequencies were as follows: A = 0.249896, C = 0.247227, G = 0.266396, and T = 0.236482; substitution rates AC = 1.541329, AG = 4.043212, AT = 1.269617, CG = 1.398831, CT = 9.131101, and GT = 1.000000; proportion of invariable sites (I) = 0.563654; and gamma distribution shape parameter (α) = 0.661958. Bayesian analysis showed similar topologies with the RAxML analysis (Figure 2 and Figure S2); therefore, only the BI tree is presented herein (Figure 2). Bayesian inference yielded 11,202 post-burn-in trees, with convergence achieved at a stop value of 0.009957.

The phylogenetic analyses (ML/BI) strongly supported the monophyly of 11 *Lophiostomataceae* genera, with high statistical support (100% ML/1.00 BPP). A total of 88 strains of *Lophiostoma* formed a monophyletic clade with 75% ML support (Figure 2). *Lophiostoma multiforme* (CCMJ 13069 and CCMJ 13070) formed a well-supported monophyletic lineage with 88% ML and 0.99 BPP values (Figure 2). *Lophiostoma montanae* (CCMJ 13067 and CCMJ 13068) grouped with *L. montanae* (MFLUCC 16-0999, UESTCC 23.0038, and UESTCC 23.0039) with 0.99 BPP values.

### 3.2. Taxonomy

#### 3.2.1. *Alloleptosphaeria xanthoceratis* R. Xu and Y. Li, sp. nov. (Figure 3)

MycoBank Number: 858873

Etymology: referring to the host plant genus *Xanthoceras* (*Sapindaceae*).

Holotype: HMJAU 60190

Description: Saprobic on dead stems of Xanthoceras sorbifolium. Sexual morph: Undetermined. Asexual morph: *Conidiomata* 131–175 × 110–200 µm ($\overset{-}{x}$ = 151 × 158 µm, n = 5), pycnidial, solitary to aggregated in small groups, semi-immersed in host substrate, black, elongate, subglobose, without a distinct ostiole. *Ostioles* absent. *Conidiomatal wall* multilayered, thick, 12–30 µm wide, comprising 3–4 layers of scleroplectenchymatous tissue, pale brown to brown, arranged in *textura angularis*. *Conidiophores* reduced to conidiogenous cells. *Conidiogenous cells* hyaline, phialidic, discrete, determinate, 9.8–22 × 1.8–3 µm ($\overset{-}{x}$ = 15 × 2.5 µm, n = 20), arising from inner wall layers. *Conidia* 3.6–6.4 × 2–6.4 µm ($\overset{-}{x}$ = 4.8 × 2.6 µm, n = 40), aseptate, hyaline, smooth-walled, guttulate, subcylindrical to narrowly ellipsoid, apex obtuse, base truncate.

Culture characteristics: Colonies reach 2 cm in diameter after 14 days at 25 °C. Colonies are dense, round, milky-white central domes, transitioning to gray toward the periphery, creeping hyphae margins irregular with pale brown; reverse white centrally, becoming dark brown peripherally.

Material examined: CHINA. Jilin Province: Changchun city, on dead stems of *Xanthoceras sorbifolium*. 16 October 2021, Rong Xu, HMJAU 60190 (holotype); living culture, CCMJ 13066.

GenBank numbers: ITS: PP151694; LSU: PP153449; SSU: PV569760; tub2: PV670045.

Notes: A BLASTn search of the ITS region revealed that our strain shares 96.41% similarity with *A. iridicola* (CBS 143395, NR_159068). The tub2 sequence is 84.92% similar to *Leptosphaeria zhaotongensis* (HKAS:124664, OP476695) with 94% query cover. In our phylogenetic study, *Alloleptosphaeria xanthoceratis* is phylogenetically closely related to three other *Alloleptosphaeria* species. To date, only *A. iridicola* has been reported to produce asexual morph within this genus [39]. Crous et al. [39] initially described *Subplenodomus iridicola* as the causative agent of Iris leaf spots in England, and it was later transferred to *Alloleptosphaeria* based on molecular evidence [40]. Morphologically, *Alloleptosphaeria xanthoceratis* is distinguished from *A. iridicola* by its larger conidiogenous cells (9.8–22 × 1.8–3 µm vs. 4–7 × 4–6 µm). We therefore formally describe it as a new species.

**Figure 3:**
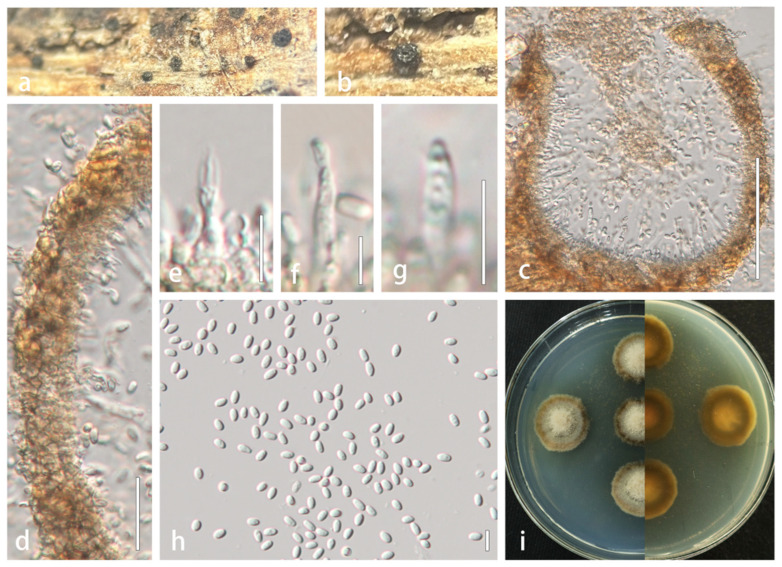
*Alloleptosphaeria xanthoceratis* (HMJAU 60190, **holotype**). (**a**,**b**) Conidiomata developing on natural substrate. (**c**) Longitudinal section through conidioma. (**d**) Wall of conidioma. (**e**–**g**) Conidiogenous cells and conidia. (**h**) Mature conidia. (**i**) Colony morphology on PDA. Scale bars: (**c**) = 100 µm; (**d**) = 20 µm; (**e**–**g**) = 10 µm; (**h**) = 5 µm.

#### 3.2.2. *Lophiostoma montanae* (Phukhams., Sue, and K.D. Hyde) Andreasen, Jaklitsch, and Voglmayr, *Persoonia* 46: 259 (2021) [41] = *Sigarispora montanae* Phukhams. Sue et al., *Fungal Diversity* 102: 55 [42], (Figure 4)

Index Fungorum number: IF557124; Facesofungi number: FoF 07295

Description: *Saprobic* on dead stems of *Xanthoceras sorbifolium*. Sexual morph: *Ascomata* dark brown to black, solitary, scattered, semi-immersed, globose, 186–350 × 160–278 μm ($\overset{-}{x}$ = 260 × 216 μm, n = 5), rough-walled, apex partially carbonaceous forming a clypeate structure, ostiolate, coriaceous. *Ostioles* central, crest-like at apex, 65–93 × 39–57 μm ($\overset{-}{x}$ = 76.1 × 49.6 μm, n = 5), elongated and laterally compressed, filled with hyaline periphyses, irregular wall. *Peridium* composed of 6–8 layers of thick-walled, brown to dark brown cells of *textura angularis*, broader at the apex and gradually tapering toward the base, 22–46 μm wide ($\overset{-}{x}$ = 33 μm, n = 20). *Hamathecium* 1–1.9 µm ($\overset{-}{x}$ = 1.6 μm, n = 20), densely arranged, hyaline, septate, frequently branched, and anastomosing within a gelatinous matrix. *Asci* bitunicate, fissitunicate, 8-spored, broad cylindrical to clavate, 102–130 × 9–14 µm ($\overset{-}{x}$ = 118 × 12 µm, n = 20), short pedicellate (pedicel furcate), apically rounded with distinct ocular chamber. *Ascospores* biseriate and partially overlapping, fusiform with attenuated ends, initially hyaline, becoming yellowish-brown at maturity, 16–29 × 4.5–6.8 µm ($\overset{-}{x}$ = 21.1 × 5.6 µm, n = 50), 3–5 transversely septa, constricted at the septa, cells above medial septum swollen, wall smooth, guttulate, surrounded by persistent mucilaginous sheath (3–5 μm) extending as polar appendages. Asexual morph: Undetermined.

Culture characteristics: Colonies on PDA reaching 30 mm in diameter after 35 days at 25 °C. Cultures from above are circular, flat, thick and dense, umbonate, entire edge, grayish white; reverse is cream-colored marginally, gradually darkening to brown at the middle region with a distinct red-brown central disc.

Material examined: CHINA. Jilin Province: Changchun city, Jinyue district, on dead stem of *Xanthoceras sorbifolium*, 10 June 2022, Rong Xu, XR36.1 (HMJAU 64835), living culture CCMJ 13067; XR36.2 (HMJAU 64836), living culture CCMJ 13068. 

GenBank numbers: CCMJ 13067: ITS = PV569791, LSU = PV569911, SSU = PV569764, *tef*1-α = PV670048, *rpb*2 = PV670046. CCMJ 13068: ITS = PV569792, LSU = PV569912, SSU = PV569765, *tef*1-α = PV670049, *rpb*2 = PV6700467.

Host: *Xanthoceras sorbifolium* (this study), *Clematis montana*, *Paeonia suffruticosa*.

Distribution: Jilin Province (this study), Yunnan Province [42], Sichuan Province [14], China.

**Figure 4:**
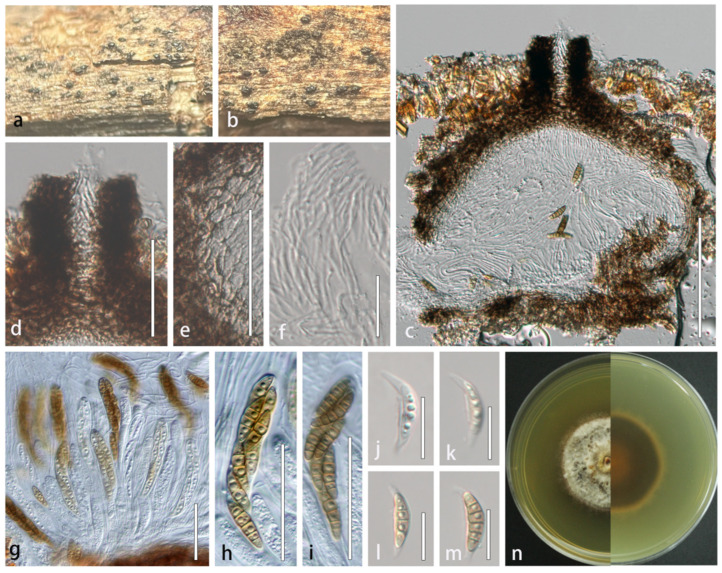
*Lophiostoma montanae* (HMJAU 64835). (**a**,**b**) Ascomata developing on natural host substrate. (**c**) Longitudinal section through ascomata. (**d**) Ostiole. (**e**) Partial peridium wall. (**f**) Anastomosing pseudoparaphyses in hamathecium. (**g**–**i**) Asci. (**j**–**m**) Ascospores. (**n**) Colony morphology on PDA. Scale bars: (**c**) = 100 μm; (**d**,**e**,**g**–**i**) = 50 μm; (**f**,**j**–**m**) = 20 μm.

Notes: *Lophiostoma montanae* was originally described by Phukhamsakda et al. [42] from dead leaves of *Clematis montana* in China, characterized by its distinctive ascomata featuring ostioles with crest-like apices. Our new isolates align morphologically with this species and form a fully supported clade (100% ML/1.00 BPP) with *L. montanae* in phylogenetic analyses (Figure 2 and Figure 4). Thus, the isolates are identified as *L. montanae*, representing the first record of this species in *X. sorbifolium* and expanding its known host range in China.

#### 3.2.3. *Lophiostoma multiforme* R. Xu and Y. Li, sp. nov. (Figure 5)

MycoBank number: 858874

Etymology: referring to its multiform of ascospores. 

Holotype: HMJAU 64837. 

Description: *Saprobic* on dead stems of *Xanthoceras sorbifolium*. Sexual morph: *Ascomata* 223–352 × 187–351 μm ($\overset{-}{x}$ = 226 × 259 μm, n = 5), black, solitary, superficial, scattered or aggregated in small groups, globose to subglobose, coriaceous. *Ostioles* 80–100 μm × 20–30 μm ($\overset{-}{x}$ = 90 × 25.4 μm, n = 5), slit-like, central. *Peridium* wider at the apex, 31–65 μm ($\overset{-}{x}$ = 43 μm, n = 20), attenuating toward the base, distinctly layered, comprising 6–8 cell layers; outer layers composed of reddish brown to dark brown, thick-walled cells of *textura angularis*, gradually transitioning inward to paler, more elongated cells of *textura prismatica*. *Hamathecium* composed of filamentous, densely branched, septate pseudoparaphyses, 1–1.7 μm wide, embedded in a gelatinous matrix, extending between and surpassing the asci. *Asci* 51–102 × 8–12 μm ($\overset{-}{x}$ = 81.9 × 10 μm, n = 30), bitunicate, fissitunicate, 8-spored, cylindrical to clavate, apex rounded with a distinct ocular chamber, base tapering to a short furcate pedicel. *Ascospores* 16–23 × 4–7 μm ($\overset{-}{x}$ = 20.1 × 5.5 μm, n = 50), 1–2-seriate, ellipsoidal to fusiform, gradually tapering toward both ends, (0-)1–2 transversely septa, initially hyaline, becoming dark brown at maturity, guttulate, smooth-walled, surrounded by a distinct mucilaginous sheath. Asexual morph: Undetermined.

Culture characteristics: Colonies on PDA attaining 30 mm diam after 10 days at 25 °C, circular with undulate margins, flat, moderately dense, greenish brown at the center, transitioning gradually to cream-colored at the periphery, with a distinct radiate pattern.

Material examined: CHINA. Hebei Province: Handan city, Qiu county, forest farm in Liangerzhuang Town, on dead stem of *Xanthoceras sorbifolium*, 9 August 2022, Rong Xu, XR98.1 (HMJAU 64837, holotype), ex-type living culture CCMJ 13069; XR98.2 (HMJAU 648838, isotype), ex-isotype living culture CCMJ 13070.

GenBank numbers: CCMJ 13069: ITS = PP151705, LSU = PP153460, SSU = PV569761, *tef*1-α = PV670043, *rpb*2 = PV670041. CCMJ 13070: ITS = PP151706, LSU = PP153461, SSU = PV569762, *tef*1-α = PV670044, *rpb*2 = PV670042.

Notes: The new collections fit well with the generic concept of *Lophiostoma* in having crest-like apices ascomata. In the phylogenetic analyses, two strains of *L. multiforme* (CCMJ 13069 and CCMJ 13070) are distinct from extant species in *Lophiostomataceae* and clustered with the strain of *L. heterospora* (AFTOL-ID 1036) with 88% ML/0.99 BPP support (Figure 2). A BLASTn analysis in GenBank revealed that the ITS sequence of CCMJ 13069 showed the highest similarity (92.77%) to *L. compressum* (MAL02, MW759267), while its LSU sequence most closely matched *Guttulispora crataegi* (MFLUCC 13-0442, NG_059563) with 97.87% similarity.

*Lophiostoma multiforme* differs from *L. heterospora* in having smaller ascomata (223–352 × 187–351 vs. 275–440 × 220–275 μm), smaller asci (51–102 × 8–12 vs. 70–110 × 15–23 μm), smaller ascospores (16–23 × 4–7 vs. 27–35 × 5–6 μm), and less septate (0–2 transversely septa vs. 8–10 transversely septa) [43]. Additionally, the ascospores of *L. heterosporum* are hyaline, while those of *L. multiforme* are initially hyaline and become dark brown at maturity. Based on morphological characteristics and multi-locus phylogenetic analyses, we propose *Lophiostoma multiforme* as a novel species.

**Figure 5:**
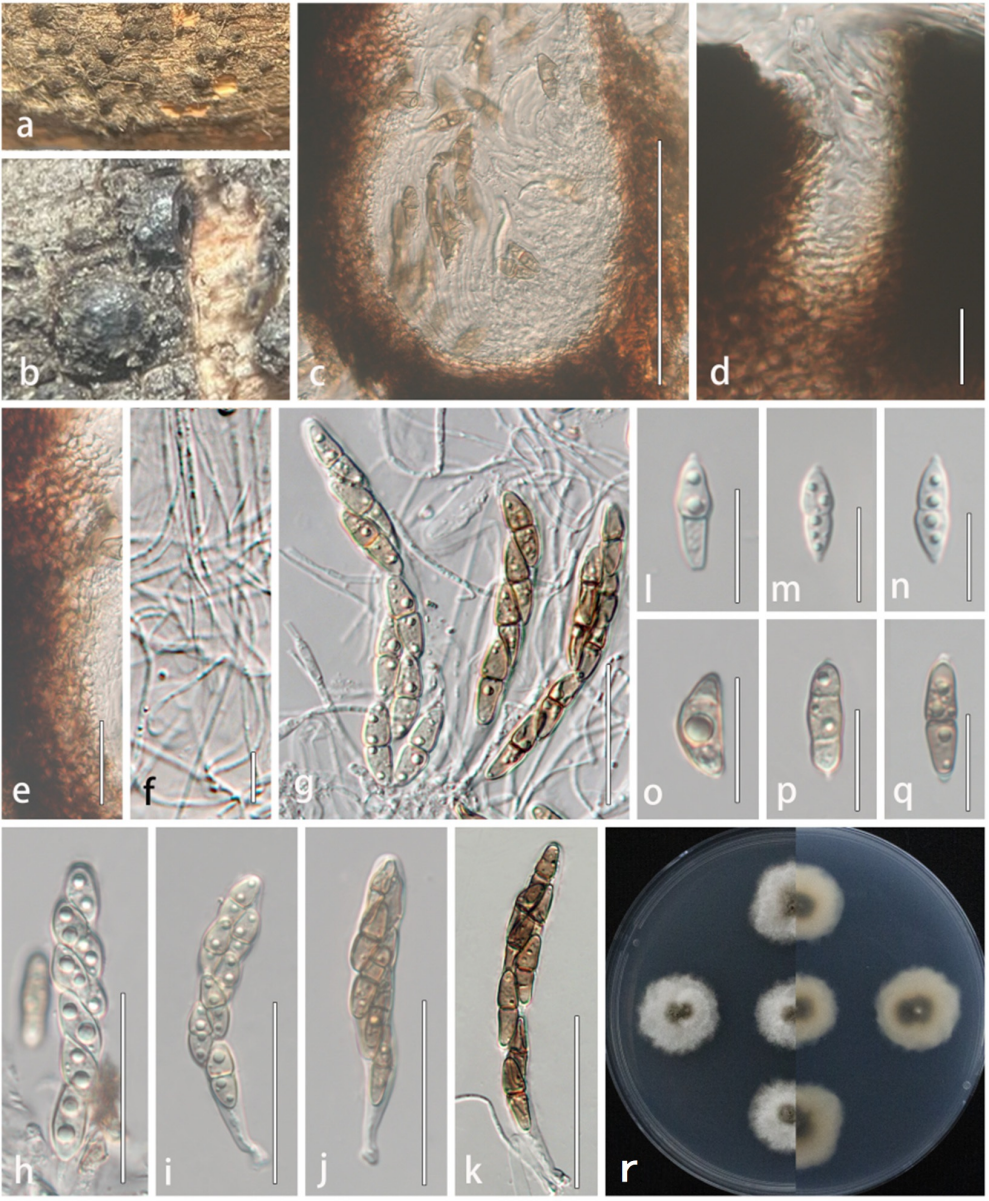
*Lophiostoma multiforme* (HMJAU 64837, **holotype**). (**a**,**b**) Ascomata developing on natural host substrate. (**c**) Longitudinal section through ascomata. (**d**) Ostiole. (**e**) Partial peridium wall. (**f**) Anastomosing pseudoparaphyses in hamathecium. (**g**–**k**) Asci. (**l**–**q**) Ascospores. (**r**) Colony morphology on PDA. Scale bars: (**c**) = 100 μm; (**d**–**e**,**l**–**q**) = 20 μm; (**f**) = 10 μm; (**g**–**k**) = 50 μm.

## 4. Discussion

*Pleosporales* was formally defined as an order by Luttrell and Barr (1987) [9]. Due to the polyphyly of traditional morphological groupings, the order has undergone extensive reorganization and significant changes in circumscription [9]. Recent taxonomic studies on *Pleosporales* have significantly refined its classification through integrative approaches combining morphology, multi-locus phylogenetics, and phylogenomic analyses [7,8,44,45,46]. The application of high-throughput sequencing is helpful for the classification and identification of controversial species [45,47]. Despite these advances, the taxonomic delineation of many species remains unresolved. A robust framework integrating evolutionary genomics and functional ecology is imperative to clarify phylogenetic relationships and ecological diversification within this economically and ecologically pivotal fungal clade.

*Alloleptosphaeria* was established by Ariyawansa et al. [48] to include the single species *A. italica*, isolated from dead stems of *Clematis vitalba* in Italy. This genus species is characterized by semi-immersed to erumpent ascomata, papillate ostiole, reddish brown to dark brown pseudoparenchymatous cells present in the thin-walled peridium, septate, cellular pseudoparaphyses, cylindric-clavate asci, and hyaline to brown ascospores that have transverse or longitudinal septa or have transverse and longitudinal septa together [39,40,42,48,49]. The asexual morph has been documented only from *A. iridicola* [39]. Currently, there are four epithets (*Alloleptosphaeria clematidis* Phukhams. and K.D. Hyde; *A. iridicola* (Crous and Denman) Voglmayr; *A. italica* Wanas., Camporesi, Ariyaw. and K.D. Hyde; and *A. shangrilana* Thiyagaraja, Tennakoon and K.D. Hyde) listed in Species Fungorum (2022) under this genus. In the present study, *Alloleptosphaeria xanthoceratis* groups with other *Alloleptosphaeria* species with strong support (Figure 1) and differs from known asexual morphs in the genus by its larger conidiogenous cells (9.8–22 × 1.8–3 µm vs. 4–7 × 4–6 µm). Both asexual morph species of *Alloleptosphaeria* were discovered in temperate regions [39], suggesting these areas may be favorable habitats for asexual members of this genus.

The genus *Lophiostoma* (type genus of *Lophiostomataceae*) was established by Cesati and De Notaris (1863), with *L. macrostomum* designated as its type species [8,50]. Species of *Lophiostoma* are characterized by laterally compressed or crest-like ascomatal apices, typically occurring as saprobes on decaying plant material in both aquatic and terrestrial environments [41,51,52]. *Lophiostoma* has been subjected to a number of revisions since its introduction [50,51,52,53]. Andreasen et al. [41] proposed the synonymization of 14 genera with *Lophiostoma* based on multi-gene phylogenetic analysis using ITS, LSU, *tef*1-α, and *rpb*2 markers. A new species, *Lophiostoma multiforme* sp. nov., and a new record of *L. montanae* are described in this study based on their morphological characteristics and phylogenetic analysis. *Lophiostoma montanae* has been documented in Yunnan and Sichuan Provinces, China, associated with *Clematis montana* and *Paeonia suffruticosa*, respectively [14,42]. Our isolate was isolated from *Xanthoceras sorbifolium* in Jilin Province. Subtle variations in morphology were observed among these strains, suggesting that host substrates may influence fungal development. The newly identified strain contributes additional molecular data to the genus *Lophiostoma*. 

Current estimates suggest 2.2–3.8 million fungal species exist worldwide, yet approximately 150,000 (3.5–7%) are formally described [1,54,55]. As the largest *Ascomycota* order, *Pleosporales* has a global distribution [7,8,9]. However, studies of microfungi in *X. sorbifolium* are very scattered, and there are a lot of fungal species that remain to be discovered [19,20,21,22]. Our results emphasize that pleosporalean fungi associated with *X. sorbifolium* are yet to be properly studied. Further research integrating multi-gene phylogenetic analysis, morphological characterization, and genomic study is essential to elucidate the diversity of microfungi associated with *X. sorbifolium* and their co-evolutionary interactions.

## Figures and Tables

**Table 1:** PCR primers utilized for amplification and sequencing in the investigation.

Loci	Primer Pair Forward/Reverse	Sequence (5′–3′)	Reference
ITS	ITS5/ITS4	GGAAGTAAAAGTCGTAACAAGG TCCTCCGCTTATTGATATGC	[26]
LSU	LR0R/LR5	ACCCGCTGAACTTAAGC ATCCTGAGGGAAACTTC	[27]
SSU	NS1/NS4	GTAGTCATATGCTTGTCTC CTTCCGTCAATTCCTTTAAG	[26]
*tef*1-α	2218F/983R	GCYCCYGGHCAYCGTGAYTTYAT ATGACACCRACRGCRACACRGTYTG	[28]
*rpb*2	fRPB2-5F/fRPB2-7cR	GAYGAYMGWGATCAYTTYGG CCCATRGCTTGYTTRCCCAT	[29]
*tub*2	T1/Bt2b	AACATGCGTGAGATTGTAAGT ACCCTCAGTGTAGTGACCCTTGGC	[30]

## Data Availability

All sequences generated in this study were submitted to GenBank.

## Supplementary Materials

The following are available online at https://www.mdpi.com/article/10.3390/microorganisms13061296/s1, Figure S1: Phylogram of *Leptosphaeriaceae* generated from maximum likelihood analysis based on combined ITS, LSU, SSU, and *tub*2 sequence data. Figure S2: Phylogram of *Lophiostomataceae* generated from maximum likelihood analysis based on combined ITS, LSU, SSU, *tef*1-α, and *rpb*2 sequence data.
