# Supplementary material for: Additions to Pleosporalean Taxa Associated with Xanthoceras sorbifolium from Jilin and Hebei, China
Source: Microorganisms. 2025 May 31;13(6):1296. doi: 10.3390/microorganisms13061296 (PMC12195281; doi:10.3390/microorganisms13061296)
Supplement: Supplementary file 1 [file microorganisms-13-01296-s001.zip › microorganisms-3657159-supplementary.pdf]

SUPPLEMENTARY MATERIAL FOR

Additions to Pleosporalean Taxa Associated with *Xanthoceras sorbifolium* from Jilin and Hebei, China

Wenxin Su[1], Ranagul·Tieliwadi[1], Wenying Su[1,3], Xiao Li[1*] and Rong Xu[1,2*]

Rong Xu 1,2 and Yu Li 1,*

1 Engineering Research Center Edible and Medicinal Fungi, Ministry of Education, Jilin Agricultural University, Changchun 130118, China; zyxurong66@126.com
2 School of Food Science and Engineering, Yangzhou University, Yangzhou 225127, China
* Correspondence: yuli966@126.com

Figure S1: Phylogram of *Leptosphaeriaceae* generated from maximum likelihood analysis based on combined ITS, LSU, SSU, and *tub*2 sequnence data.
Figure S2: Phylogram of *Lophiostomataceae* generated from maximum likelihood analysis based on combined ITS, LSU, SSU, *tef*1-α and *rpb*2 sequnence data.

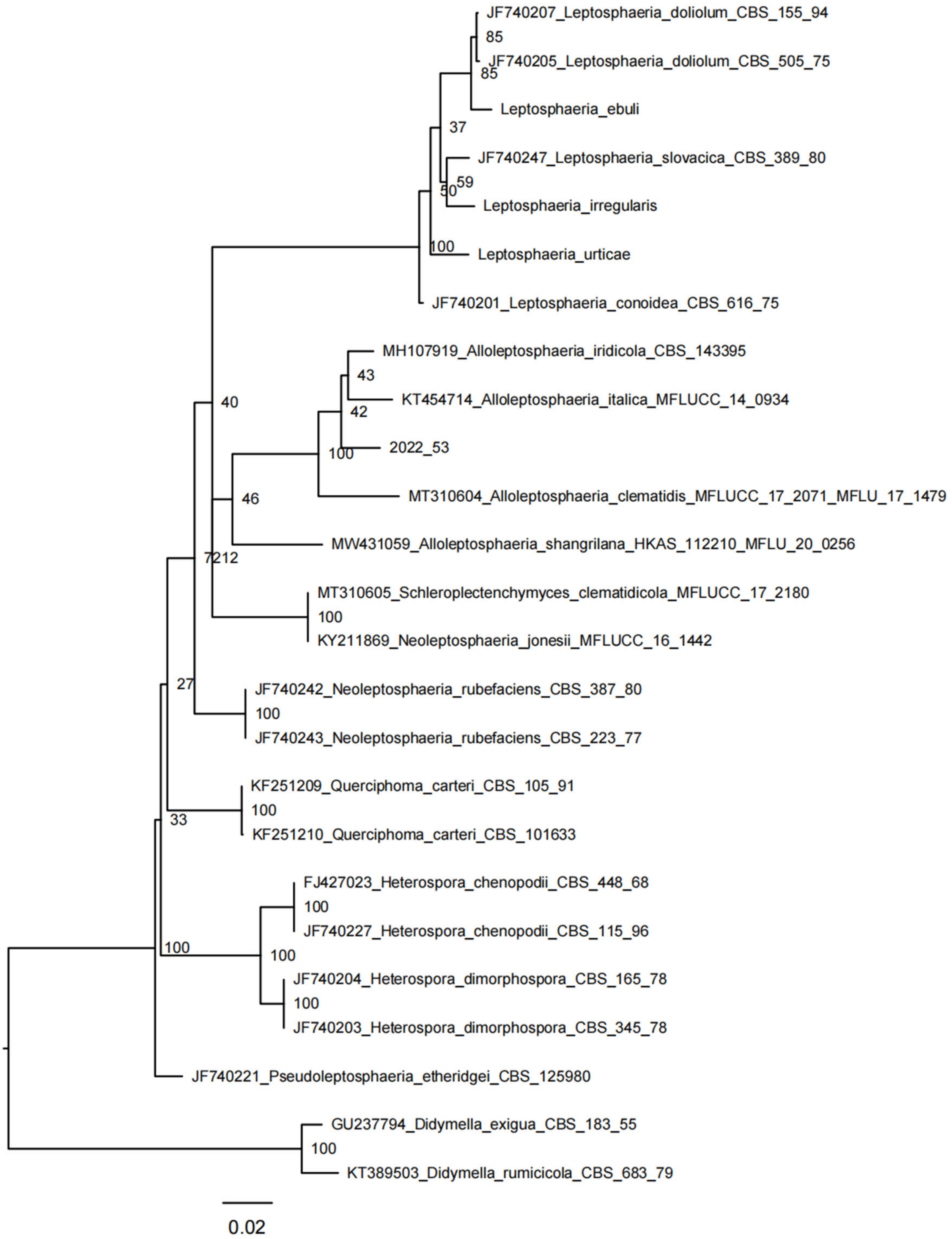

Figure S1: Phylogram of *Leptosphaeriaceae* generated from maximum likelihood analysis based on combined ITS, LSU, SSU, and *tub*2 sequence data.

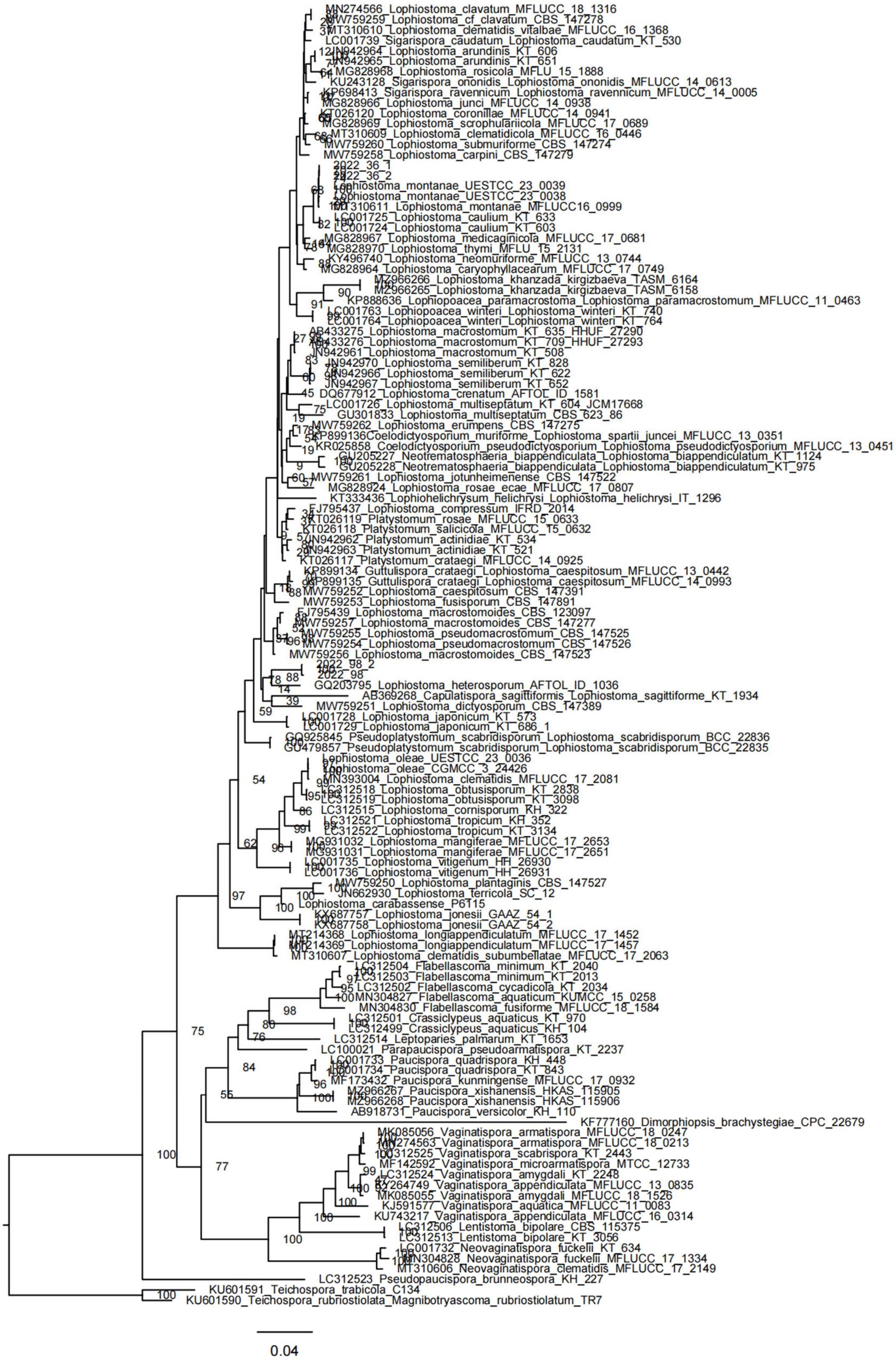

Figure S2: Phylogram of *Lophiostomataceae* generated from maximum likelihood analysis based on combined ITS, LSU, SSU, *tef*1-α and *rpb*2 sequnence data.
